# Turkish adaptation and psychometric testing of the Perceived Workplace Support Scale in people with inflammatory arthritis

**DOI:** 10.1093/rap/rkag066

**Published:** 2026-06-09

**Authors:** Gonca Bumin, Ezginur Gündoğmuş, Alan Tennant, Sevilay Karahan, Umut Kalyoncu, Yeliz Prior

**Affiliations:** Department of Occupational Therapy, Faculty of Health Sciences, Hacettepe University, Ankara, Turkey; Department of Occupational Therapy, Faculty of Health Sciences, Hacettepe University, Ankara, Turkey; Leeds Institute of Rheumatic and Musculoskeletal Medicine, University of Leeds, Leeds, UK; Department of Biostatistics, Faculty of Medicine, Hacettepe University, Ankara, Turkey; Department of Rheumatology, Faculty of Medicine, Hacettepe University, Ankara, Turkey; Centre for Human Movement and Rehabilitation, School of Health and Society, University of Salford, Salford, UK; Arthritis UK/MRC Centre for Musculoskeletal Health and Work, School of Health and Society, University of Salford, Salford, UK

**Keywords:** inflammatory arthritis, workplace support, Turkish adaptation, Beaton method, psychometric validation, Rasch model

## Abstract

**Objective:**

This study aimed to adapt the Perceived Workplace Support Scale (PWSS) into Turkish and evaluate its psychometric properties among individuals diagnosed with inflammatory arthritis (IA).

**Methods:**

A total of 200 employed individuals with IA (mean age = 37.9 ± 10.6 years; 66.5% female) participated in the study. The cultural adaptation followed Beaton’s method, including forward translation, synthesis, back-translation, expert committee review and cognitive debriefing. Construct validity was assessed using Rasch analysis. Internal consistency was examined with Cronbach’s alpha, and test–retest reliability was evaluated in a subsample using intraclass correlation coefficients (ICCs). Concurrent validity was examined by correlating PWSS dimension scores with the Work Limitation Questionnaire–Short Form (WLQ-SF), Rheumatoid Arthritis Work Instability Scale (RA-WIS) and Work Productivity and Activity Impairment Questionnaire: General Health (WPAI: GH).

**Results:**

Rasch analysis supported the structural validity of the Turkish PWSS, showing adequate model fit after addressing local item dependencies. Cronbach’s alpha ranged from 0.59 (manager) to 0.81 (coworker). Test–retest reliability indicated fair stability, with ICCs of 0.47 (manager), 0.52 (coworker) and 0.59 (organization). These findings suggest that the scale is more appropriate for group-level comparisons rather than individual-level assessment. The scores for PWSS subdimensions showed moderate correlations with work and health measures, supporting concurrent validity.

**Conclusions:**

The Turkish version of the PWSS demonstrates potential utility for assessing perceived workplace support in individuals with IA. However, given the fair level of test–retest reliability, it should be used with caution and primarily for group-level analyses.

Key messages

- The Perceived Workplace Support Scale (PWSS) provides a measure of perceived workplace support in Turkish employees; however, its test–retest reliability is low, and it is therefore more appropriate for group-level use in research rather than individual-level assessment.
- The scale reflects meaningful associations between workplace support and job functioning outcomes.
- PWSS may help inform clinical, research and workplace efforts to support employees with chronic conditions.

## Introduction

Inflammatory arthritis (IA) is a chronic disease group that negatively affects individuals’ physical, psychological and social functionality [1]. Individuals with IA, especially in the workforce, face various difficulties in the workplace, which may negatively impact their work performance, job satisfaction and attendance [2, 3]. Social, physical and managerial support provided at the workplace is important in helping these individuals cope with work-related difficulties [4]. This concept, which is also defined as perceived organizational support (POS) in the literature, includes employees’ subjective evaluations of the level of support they receive from the workplace [5]. POS, generally conceptualized as employees’ beliefs that their organization appreciates their efforts and is concerned for their psychological health, is considered a key factor influencing the types of strategies managers may implement to promote employee well-being [6].

Traditionally, organizational support theory has emphasized the organization itself as the primary source of support for employees. However, it is important to examine not only organizational support but also other elements of support in the workplace [7]. It is also important for employees to distinguish the support they receive from the organization as a whole from the support they receive from specific components within the organization, such as supervisors and coworkers [8]. This broader, multifocal perspective has contributed to the development of organizational support theory by including these individual sources. For example, perceived supervisor support (PSS) and perceived colleague support (PCS) refer to employees’ perceptions that their managers and colleagues appreciate their efforts and are concerned about their well-being [9, 10]. Assessment scales that evaluate such factors and have been tested across a range of IA conditions can help identify how the working environment of people with IA affects work participation, provide access to practical help and work accommodations, but also help identify interventions to address workplace culture, psychological support and disability-related work issues with a focus on accessibility, and assess changes post-intervention. The Perceived Workplace Support Scale (PWSS) developed in this context consists of three subscales for manager, coworker and organizational support. Each has excellent internal consistency in working people with IA and OA [11–13]. The PWSS has a British English version and has been reported to have high internal consistency [14].

There are some scales that address these parameters separately. For organizational support, the POS scale [15] and the Perceived Institutional Support Scales [16] have been developed. For social support, there are Perceived Social Support Scales [17, 18] that assess social support from family and friends. However, none of these scales addressed these three factors together and were not developed for individuals with IA. There is no scale that directly measures the support that individuals with rheumatologic diseases receive at work in Türkiye. Therefore, this study aimed to adapt the PWSS into the Turkish language and to examine its validity and reliability in individuals diagnosed with IA. Thus, it was aimed to provide a culturally appropriate and psychometrically valid assessment tool for researchers and clinicians who want to measure the perception of workplace support.

## Methods

### Study design, participants and recruitment procedures

A total of 200 individuals who were referred to the Division of Rheumatology, Department of Internal Medicine, Hacettepe University Faculty of Medicine, and received a clinical diagnosis of IA by a rheumatologist were included in the study. Inclusion criteria were: age ≥18 years, confirmed IA diagnosis and current employment (full-time or part-time, working at least 1 day per week). In the study, calculations were performed based on a correlation coefficient of 0.32, which is the lowest reported in the literature, for the scales to be analysed for validity and reliability. In the statistical power calculation, it was determined that a total of 138 individuals were needed to achieve a 1% alpha error rate and 90% statistical power [19]. Considering the potential for missing data and participants who may be lost during retest evaluations, it was planned to include 200 individuals in the study. Individuals on medically certified sick leave were excluded. Ethical approval was obtained from the Hacettepe University Health Sciences Research Ethics Committee (decision number: 2024/01–03), and written informed consent was secured from all participants. Construct validity was assessed using Rasch analysis, which requires a minimum of 150 participants per group [20]. To enhance response variability, the target sample size was set at 200. For test–retest reliability analysis, a minimum of 79 participants was required. Participants completed the PWSS a second time after a 15-day interval, which is considered sufficient to minimize recall bias while ensuring that no substantial change in workplace support is expected [21].This sample size was calculated to detect a statistically significant difference between an expected reliability coefficient (correlation) of 0.70, which is generally considered an acceptable threshold for good reliability [22], and a background correlation of 0.45, representing a fair level of reliability that we aim to improve upon. The calculation was based on achieving 90% statistical power, which reduces the likelihood of Type II errors, and using a 1% significance level to account for multiple comparisons and ensure a more conservative test. These parameters were chosen to balance statistical rigour with practical feasibility in sample recruitment [22]. Cultural adaptation of the scale followed the methodology outlined by Beaton et al. [23].

#### Phase 1

The original English version of the scale was independently forward translated into Turkish by two native Turkish-speaking experts. One of the translators is the first author, a professor with nearly 30 years of experience in occupational therapy in Turkey. The second translator is an academic with over 5 years of experience in the field of occupational therapy, both clinically and in higher education. Both translators contributed to ensuring linguistic accuracy and conceptual fidelity in the forward translation process. The two forward translations were compared with identify discrepancies, including ambiguous expressions and inconsistencies. Through discussion and consensus, a harmonized version (T-12) was synthesized, drawing on the original scale and both translations (T1 and T2).

The T-12 version was then back-translated into English by two native English-speaking translators blinded to the original instrument. The first translator is a British Turkish professor with over 20 years of experience in occupational therapy in the UK. The second translator is a professional translator from a non-medical background, with 18 years of experience. The back-translated versions were reviewed for conceptual and semantic equivalence with the original scale. Following the reconciliation of discrepancies, a pre-final version was produced, ensuring linguistic and conceptual fidelity to the source instrument. Additionally, the face validity of the preliminary version was assessed by two occupational therapists, who evaluated each item for clarity, relevance and conceptual consistency with the source instrument. Following the translation and expert review process, cognitive debriefing interviews were conducted with 30 people diagnosed with IA to assess the clarity, comprehensibility, and cultural relevance of each item. All participants reported that the items were clear and relevant, and no modifications were required based on their feedback.

#### Phase 2

To assess the concurrent validity of the PWSS, participants were asked to complete the Turkish version of the PWSS along with a battery of additional questionnaires measuring related work and health constructs, as detailed below.

##### Demographic information form

Data were collected on participants’ sociodemographic and clinical characteristics, including age, sex, height, weight, BMI, diagnosis, time since diagnosis, duration of symptoms and current employment status (part-time or full-time). Work-related variables such as average weekly working hour, size of the workplace and the physical demands of the job were also documented. In addition, information on participants’ educational attainment, marital status (married or single) and current medication use was recorded.

##### Perceived Workplace Support Scale

The PWSS was developed by Gignac in Canada [11, 12]. It consists of a 19-item questionnaire with four items measuring perceived management support, eight items measuring perceived coworker support and seven items measuring company, business or organization support. The items are rated on a 5-point scale (1 = strongly disagree and 5 = strongly agree). Cronbach’s alpha for the British English version of the test items are 0.87, 0.87, 0.83 and 0.89 for RA, axSpA, OA and FM, respectively [14]. In the original Canadian longitudinal study by Gignac, Cronbach’s alpha for the managerial support subscale was 0.87 at time 1 and 0.86 at time 2, time 3 and time 4. For the coworker support subscale, Cronbach’s alpha values were 0.87, 0.89, 0.90, and 0.91 at time 1, time 2, time 3 and time 4, respectively [11].

##### Work Limitation Questionnaire–Short Form

The Work Limitation Questionnaire–Short Form (WLQ-SF) evaluates the extent to which physical or emotional health problems experienced over the past 2 weeks have interfered with work performance [24]). It comprises two items for each of four domains: time management, physical demands, mental-interpersonal demands and output demands. Each item is rated on a 6-point Likert scale (1 = all of the time; 6 = does not apply to my job). Higher scores on the WLQ-SF indicate greater work limitations, reflecting more frequent interference of health problems with work performance. Conversely, lower scores reflect fewer perceived limitations and better work functioning. The scale requires approximately 10–15 min to complete. The Turkish version has demonstrated acceptable internal consistency, with a Cronbach’s alpha of 0.83 [25].

##### Rheumatoid Arthritis Work Instability Scale

The Rheumatoid Arthritis Work Instability Scale (RA-WIS) is a 23-item, dichotomous (true/false) instrument designed to assess the degree of mismatch between functional capacity and job demands in individuals with rheumatoid arthritis (RA) by examining disease-related impacts on work performance [26]. Higher scores indicate greater work instability risk; lower scores reflect better alignment between abilities and job demands. As no formally validated Turkish version of the RA-WIS was available, the scale was translated into Turkish for the purposes of this study. The Cronbach’s alpha value for the scale used in this study is 0.918 for the participants.

##### Work Productivity and Activity Impairment Questionnaire: General Health Version 2.0

The Work Productivity and Activity Impairment Questionnaire: General Health (WPAI: GH) consists of six core questions and associated sub-items that assess the impact of health problems on work productivity and daily activities over the past 7 days. It quantifies four outcome domains—absenteeism (work time missed), presenteeism (reduced productivity while at work), overall work productivity loss and activity impairment—expressed as percentages, with higher scores indicating greater impairment [27]. The intraclass correlation coefficient (ICC) value of the Turkish adaptation of the test is 0.913, and the Cronbach’s alpha value is 0.662 [28].

##### Health Assessment Questionnaire

The HAQ is a 20-item instrument that measures physical functioning and disability in individuals with RA [26]. Higher HAQ scores indicate greater disability and reduced physical functioning, whereas lower scores reflect better functional status. The Turkish version has been validated and exhibits excellent reliability, with a Cronbach’s alpha of 0.97 [29].

##### Rheumatoid Arthritis Impact of Disease

The Rheumatoid Arthritis Impact of Disease (RAID) assesses the overall impact of RA on individuals’ daily life [30]. It includes seven numeric rating scales (NRS), each ranging from 0 to 10, capturing domains such as pain, fatigue and physical function. The total score is the sum of the individual domain scores. Higher scores indicate greater disease impact. The Turkish adaptation has demonstrated high internal consistency, with a Cronbach’s alpha of 0.93 [31].

In addition to standardized scales, health-related perceptions were assessed using NRS to evaluate participants’ confidence in working (1–10), mood (1–10), severity of hand pain (1–10) and overall health status (1–5).

### Statistical analysis

All statistical analyses were performed using IBM SPSS Statistics for Windows Version 23.0 (IBM Corp. Armonk, NY, USA), except Rasch analyses, which were performed using RUMM2030 software. Numerical variables are expressed as mean ± S.D. and median (25th–75th percentile), while categorical variables are summarized as frequencies and percentages. Normality of numerical variables was assessed using graphical methods and the Kolmogorov–Smirnov test. Due to non-normal distributions observed in some outcome variables, non-parametric tests (Spearman rank correlation) were used in correlation analyses. Correlation coefficients were interpreted according to Cohen’s classification: values around 0.10–0.29 were considered low, 0.30–0.49 were considered moderate, and 0.50 and above were considered strong correlations [32]. The internal construct validity of the PWSS was assessed by Rasch analysis [33]. This included examining item and person fit residuals, Chi-square statistics, irregular thresholds, local item dependencies (item residual correlation >0.2) and unidimensionality (with independent t-tests less than 5% significant indicating acceptable unidimensionality). Differential item functioning (DIF) was assessed for various contextual variables such as sex, age, diagnosis, BMI, medication use and retest time point [20]. When necessary, item groupings (testlets or superitems) were applied to accommodate local dependencies and improve model fit [33].

Internal consistency was assessed using Cronbach’s alpha coefficients and the Person Separation Index (PSI), with values ≥0.70 considered acceptable [21]. Test–retest reliability was assessed using the ICC, calculated with a two-way mixed-effects model based on absolute agreement. This model was selected because the same instrument was administered to the same participants at both time points, and the aim was to evaluate absolute agreement between repeated measurements rather than consistency alone [34]. ICC values were interpreted according to Cicchetti (1994) as follows: <0.40 = poor, 0.40–0.59 = fair, 0.60–0.74 = good and ≥0.75 = excellent reliability [35]. Additionally, the standard error of measurement (SEM) and the smallest detectable difference (SDD = 1.96 × √2 × SEM) were calculated for each subscale, as recommended for evaluating measurement error and sensitivity to change in patient-reported outcome measures [23].

Floor and ceiling effects were examined for each subscale. An effect was considered present if more than 15% of participants received the lowest or highest possible value, as recommended in the scale development literature [21].

## Results

Participants in phase 1 of the study (*n* = 30), who took part in the cognitive debriefing interviews, are described in Table 1. All participants rated the items as highly relevant. The final version of the instrument was confirmed without any modifications in item wording. Phase 2 of the study included 200 participants with a mean age of 37.9 years (SD = 10.6), of whom 66.5% were female. Descriptive analysis of individual rating scales revealed that participants reported a moderately high level of confidence in their work (mean = 7.3 ± 2.9), indicating a relatively positive self-perception of work ability. Mood scores were moderate (mean = 4.6 ± 2.7), suggesting some degree of emotional burden. Hand pain severity was also reported as moderate (mean = 4.3 ± 3.2), consistent with the nature of IA. The average overall health status score was 2.9 ± 0.8 on a 5-point scale, indicating a generally fair perception of health among participants. These findings provide additional context on the participants’ physical and psychological status. Additional demographic characteristics are provided in Table 1.

**Table 1:** Demographic information of phase 1 and phase 2 participants.

		Phase 1 (*n* = 30)		Phase 2 (*n* = 200)	
		Mean ± SD	Min–max	Mean ± SD	Min–max
Age (year)		43.0 ± 12.3	23–64	37.9 ± 10.6	19–66
Height (cm)		160.7 ± 31.2	152–188	168.6 ± 8.7	145–190
Weight (kg)		72.6 ± 13.0	53–105	70.3 ± 12.0	48–105
BMI (kg/m²)		26.4 ± 4.5	20.9–39.1	24.7 ± 3.7	18.0–39.1
Duration of diagnosis (years)		11.4 ± 10.7	1–35	9.2 ± 8.0	1–35
Duration of symptoms (years)		12.7 ± 10.6	1–36	10.6 ± 8.3	1–36
Average working hour per week		39.1 ± 11.7	8–60	42.3 ± 9.3	10–72
Confidence in working		6.56 ± 3.4	0–10	7.3 ± 2.9	0–10
Mood		4.7 ± 2.7	0–10	4.6 ± 2.7	0–10
Overall health status		3.0 ± 0.8	1–5	2.9 ± 0.8	1–5
Severity of hand pain		3.6 ± 3.1	0–10	4.3 ± 3.2	0–10
		Frequency	Percent	Frequency	Percent
Gender	Female	22	73.3	133	66.5
	Male	8	26.7	67	33.5
Diagnosis	RA	21	70	130	65
	axSpA	9	30	49	24.5
	U-SpA			1	0.5
	PsA			20	10.0
Workplace size	0–50 person	15	50	75	37.5
	50–100 person	3	10	40	20.0
	100+	12	40	85	42.5
Employment status	Full time	27	90	187	93.5
	Part-time	3	10	13	6.5
Physical demands of the job	Low	10	33.3	45	22.5
	Moderate	15	50	96	48.0
	High	5	16.7	59	29.5
Education level	Primary school	1	3.3	7	3.5
	Secondary school	2	6.7	7	3.5
	High school	5	16.7	23	11.5
	University	19	63.3	129	64.5
	Postgraduate	3	10	34	17.0
Marriage status	Married	19	63.3	134	67.0
	Single	11	33.7	66	33.0
Medication type	None			6	3.0
	NSAID	1	3.3	6	3.0
	Steroid			9	4.5
	Single DMARD	20	66.7	101	50.5
	Combination DMARD	4	13.3	34	17.0
	Neuropathic analgesic			1	0.5
	Biologic/biosimilar	1	3.3	15	7.5
	Steroids, NSAIDs			1	0.5
	NSAIDs, analgesic	4	13.3	27	13.5

The category ‘u-axSpA’ refers to undifferentiated spondyloarthritis, describing participants with features of spondyloarthritis who did not meet classification criteria for psoriatic or axial SpA. The axial SpA category includes both non-radiographic axial SpA (nr-axSpA) and radiographic axial SpA (r-axSpA, equivalent to ankylosing spondylitis), according to the Assessment of SpondyloArthritis International Society (ASAS) criteria.

SD, standard deviation; NSAID, non-steroidal anti-inflammatory drug; DMARD, disease-modifying antirheumatic drugs.

The results of the work and health assessment conducted in phase 2 are demonstrated in Table 2.

**Table 2:** Descriptive statistics for the phase 2 participants’ work and health measures scores (*n* = 200).

	Mean ± SD	Median [25th to 75th percentile]
PWSS M total	13.26 ± 3.18	13 [11–16]
PWSS C total	28.79 ± 5.01	30 [26–32]
PWSS O total	23.05 ± 5.95	24 [19–28]
WLQ-TM	3.16 ± 1.16	3 [2.5–4]
WLQ-PD	2.45 ± 0.89	2.5 [2–3]
WLQ-MID	3.77 ± 0.87	4 [3–4.5]
WLQ-OD	3.52 ± 1.12	3.5 [3–4.5]
WLQTOTAL	12.90 ± 2.39	13 [11.5–14.5]
WLQpercentageloss	15.04 ± 4.44	14.58 [11.85–18.58]
RAWIS	12.04 ± 6.40	13 [7–17]
WPAI:GH1	13.14 ± 22.53	0 [0–18]
WPAI:GH2	44.00 ± 25.12	50 [20–60]
WPAI:GH3	34.92 ± 21.65	30 [20–50]
WPAI:GH4	48.80 ± 26.11	50 [30–70]
HAQ	13.27 ± 4.05	13 [10–17]
RAID	4.95 ± 2.23	4.8 [2.94–6.96]

PWSS, Perceived Workplace Support Scale; M, Manager; C, Co-worker; O, Organization; WLQ, Work Limitation Questionnaire; TM, time management; PD, physical demands; MID, mental-interpersonal demands; OD, output demands; RAWIS, Rheumatoid Arthritis Work Instability Scale; WPAI, Work Productivity Activity Impairment; HAQ, Health Assessment Questionnaire; RAID, Rheumatoid Arthritis Impact of Disease.

### Construct validity

Adaptation of the PWSS into the Turkish language showed acceptable fit of the various subscales to the Rasch model after accommodation of local item dependencies. There was an absence of DIF across a range of contextual factors and by language, confirming cross-cultural validity. The only weaknesses noted were the low reliability of the manager subscale, although this improved after the merger with the Company domain to create an organization subscale. Otherwise, the remaining problem was the ‘Do not agree or disagree’ category option, which consistently failed to work properly across most items, albeit marginally so.

#### Manager subscale

In the Manager subscale of four items, three had disordered thresholds. The middle response option of ‘Do not agree or disagree’ was the problem (Supplementary Data S1, available at *Rheumatology Advances in Practice* online). Local item dependency was present with an average residual correlation of −0.31, and two positively oriented items had a residual correlation of 0.276. The scale was grouped into two super items that barely fit adequately (Table 3). There was no DIF. Reliability was poor, with a PSI of 0.67. Targeting was adequate, with the mean logit of persons at 0.342, when the item mean was zero.

**Table 3:** Fit of the Perceived Workspace Support Scale in the Turkish Adaptation: calibration sample.

Diagnosis/scale	Residuals		Chi-Square		Reliability		Dimensionality	DIF
	Item	Person	Value (df)	*P*	PSI	α	% t-tests (LCI)	
Manager	0.6935	0.9661	10.2 (4)	0.037	0.67	0.59	1.05	None
Coworkers	0.3325	0.7217	5.3 (4)	0.256	0.81	0.81	6.0 (3.0)	None
Organization	4.0100	0.8491	2.38 (4)	0.666	0.79	0.66	2.0	None
Manager + organization	0.7056	1.0450	1.03 (4)	0.905	0.79	0.79	5.5 (2.5)	None
Total	0.4510	0.9783	0.55 (4)	0.968	0.84	0.84	5.0	None
Ideal values	<1.4	<1.4		>0.05	>0.70	>0.70	<5.9	None

α, Cronbach’s alpha; DIF, differential item functioning; LCI, lower confidence interval; PSI, Person Separation Index.

#### Coworkers subscale

In the Coworkers subscale of eight items, four had disordered thresholds. Once again, the middle response option of ‘Do not agree or disagree’ was the problem. Local item dependency clustered into the swapped item set, reflecting the difference between the positive and negatively orientated items. Consequently, these were grouped into two super items, where fit was adequate (Table 3). Reliability was adequate with a PSI of 0.81 and there was no DIF. However, targeting was barely adequate, with a person logit mean of 0.806.

#### Organization subscale

In the Organization subscale of seven items, all but one item had ordered thresholds (Supplementary Data S2, available at *Rheumatology Advances in Practice* online). The remaining item again compromised by the ‘Do not agree or disagree’ category. The two items with a positive orientation (i.e. un-swopped) had a residual correlation of 0.439 and were presented as one super item against the rest. Fit was adequate, and there was no DIF, with a PSI of 0.79 (Table 3). Targeting was again adequate with a mean person logit of 0.352.

#### Manager and Organization joint subtitle

Given the weakness of the manager subscales, consideration was given to an ‘Organization’ subscale, which merged the Manager and Organization subscales. Only 3 out of 11 items had ordered thresholds, with the middle category again the problem for all disordered items. Local item dependency was clustered into the two subscales, but these, presented as testlets, failed to resolve fit. Instead, an alternative item strategy was employed (i.e. items 1, 3, 5, etc. into one testlet; 2, 4, 6, etc. into the other), which showed adequate fit (Table 3). Once again, no DIF was observed, reliability (PSI) was adequate at 0.79, and targeting was satisfactory (mean persons 0.305).

In summary, for the subscale analyses, fit to the Rasch model was achieved through various strategies to accommodate local item independence, deal with the problem caused by the middle item category and the positive and negative worded items. Overall, targeting was adequate for most subscales with the exception of the coworkers subscale at logit 0.806.

#### Total score

A total score representing overall workplace support to considered. While testlet version of the three domains gave mostly adequate fit, reliability was extremely low. Consequently, an alternative item strategy was employed which gave good fit and reliability (PSI 0.84) (Table 3). Targeting was just adequate with a person mean of 0.501. This result was supported by a conditional Chi-Square test of fit with significance of *P* = 0.016. The transformation of raw scores to metrics for each subscale and total score is given in Supplementary Data S3, available at *Rheumatology Advances in Practice* online.

### Cross-cultural validity

All subscales and total score were then tested for invariance (DIF) against the original English adaptation on a pooled sample of 400 cases (Table 4). All showed adequate fit to the Rasch model and invariance across the two languages.

**Table 4:** Cross-cultural validation of PWSS.

Diagnosis/scale	Residuals		Chi-square		Reliability		Dimensionality	DIF
	Item	Person	Value (df)	*P*	PSI	α	% t-tests (LCI)	
Manager	0.3480	1.3604	17.9 (10)	0.057	0.69	0.67	2.5	None
Coworkers	0.1807	0.9542	8.9 (10)	0.541	0.80	0.82	3.8	None
Organization	1.3980	1.1361	11.6 (10)	0.309	0.88	0.88	4.0	None
Manager + organization	0.9370	1.0404	3.5 (10)	0.968	0.83	0.84	5.25 (3.1)	None
Total	0.5026	1.1179	1.40 (10)	0.999	0.87	0.87	5.25 (3.1)	None
Ideal values	<1.4	<1.4		>0.05	>0.70	>0.70	<5.9	None

α, Cronbach’s alpha; X DIF, cross-cultural differential item functioning; LCI, lower confidence interval; PSI, Person Separation Index.

### Concurrent validity

The correlation of all subscales of the scale with other scales is low or moderate (Table 5).

**Table 5:** Concurrent validity of the PWSS subdimensions with work and health measures.

	M, *P*-value	C, *P*-value	O, *P*-value
WLQTMTOT	0.238	0.205	0.072
WLQ2PDTOT	−0.058	−0.162	−0.081
WLQMIDTOT	0.159	0.154	0.010
WLQODTOT	0.320	0.282	0.097
WLQTOTAL	0.335	0.258	0.056
WLQpercentageloss	0.300	0.249	0.077
RAWIS	0.092	0.108	0.008
WPAI:GH1	0.064	0.058	0.263
WPAI:GH2	−0.145	−0.195	−0.041
WPAI:GH3	−0.181	−0.215	−0.176
WPAI:GH4	−0.031	−0.094	−0.031
HAQ	0.000	−0.110	−0.020
RAID	−0.147	−0.170	0.000

PWSS, Perceived Workplace Support Scale; M, Manager; C, co-worker; O, Organization; WLQ, Work Limitation Questionnaire; TM, time management; PD, physical demands; MID, mental-interpersonal demands; OD, output demands; RAWIS, Rheumatoid Arthritis Work Instability Scale; WPAI:GH, Work Productivity Activity Impairment General Health; HAQ, Health Assessment Questionnaire; RAID, Rheumatoid Arthritis Impact of Disease.

### Test–retest reliability

Across the three PWSS total scores, median values at the first (T1) and second (T2) administrations were very similar (Table 6). Test–retest reliability analysis was conducted in a subsample of 79 participants who completed the questionnaire at both time points. Test–retest reliability was assessed using ICCs. The results indicated fair reliability across subdimensions (ICC: 0.47 for Manager, 0.52 for Coworkers and 0.59 for Organization). The test–retest correlation between the two administrations was fair across all subdimensions. The SDD values were calculated as 3.97 for the M subdimension, 4.56 for the C subdimension and 4.79 for the O subdimension.

**Table 6:** Test–retest reliability of PWSS subdimensions.

	T1	T2	*P*	Spearman corr. coef.	ICC (2,1) (95% CI)	SEM	SDD
PWSS Total 1	13 [11–16]	12 [10–14]	0.176	0.521	0.470 (0.279–0.625)	2.06	3.97
PWSS Total 2	29 [25–31]	28 [24–30]	0.109	0.575	0.528 (0.348–0.670)	2.70	4.56
PWSS Total 3	24 [18–27]	22 [17–26]	0.026	0.605	0.593 (0.429–0.719)	2.98	4.79

ICC, intraclass correlation coefficient; SEM, standard error of measurement; SDD, smallest detectable difference.

### Floor and ceiling effect

The Turkish PWSS has no floor and ceiling effect. PWSS Manager Floor effect: 1.5%; Ceiling effect: 3%; PWSS Coworkers Floor effect: 1%; Ceiling effect: 1%; PWSS Organization Floor effect: 1.5%; Ceiling effect: 4.5%.

## Discussion

This study aimed to adapt the PWSS into Turkish and examine its psychometric properties in individuals with IA. The findings provide preliminary support for the scale’s psychometric properties confirming its potential utility in both clinical and research settings within the Turkish context. Furthermore, the results contribute to the growing body of literature highlighting workplace support as a key factor in the sustainability of employment and the quality of life for individuals with rheumatologic diseases.

The construct validity of the Turkish version of the PWSS was supported by Rasch analysis, which revealed that the three-dimensional structure (manager, coworker and organization) of the scale was preserved mainly after addressing local item dependencies. This is consistent with previous studies that have validated multidimensional workplace support measures such as ‘top management support’ [36]. However, the presence of disordered thresholds, especially in items with a neutral response category (‘Do not agree or disagree’), mirrors findings from previous research and points to a common psychometric challenge in Likert-type scales [20]. Local item dependencies were addressed by creating super-items, which improved scale fit. These findings align with research emphasizing the importance of testlet modelling in health measurement to address multidimensionality and local dependency [33].

In the current study, Cronbach’s alpha values ranged from 0.59 to 0.84 across subscales, slightly lower than those reported by Hammond et al. [14], where alpha values were consistently above 0.80. This discrepancy may be due to sample differences in employment conditions or employer and coworker expectations between British and Turkish employees with IA. The lack of clear workplace rules in Türkiye [37], competitive and comparative environment, distrust and lack of open communication, and workload and time constraints [38] may have influenced these results. In addition, the organization subscale in the Turkish version showed the highest reliability (α = 0.88), overlapping with the pattern observed in the British sample. This may be due to the collectivist structure of Türkiye, which enables Turkish employees to feel a sense of belonging and identity within the organization [39].

The Turkish PWSS subscales showed low to moderate correlations with work-related outcomes (e.g. WLQ, RA-WIS, WPAI), consistent with Hammond et al. (2023), reinforcing the important yet crucial role of perceived workplace support in predicting work engagement. Brown et al. [40] identified lack of workplace support as one of the predictors of higher presenteeism in RA, axSpA, OA and FM . In the current study, PWSS scores (particularly in the domains of manager and coworker) were moderately associated with loss of work productivity and instability, confirming the protective role of support in maintaining job performance and a moderating effect on work engagement and psychosocial outcomes in people with IA [8, 9].

The test–retest analysis demonstrated fair stability of the PWSS subdimensions over time. The ICC values observed in the present study (0.47–0.59) indicate fair test–retest stability according to commonly used interpretation guidelines. From a classical psychometric perspective, such values suggest that the PWSS is more appropriate for group-level comparisons in research contexts rather than precise monitoring of individual change over time. This pattern is not uncommon for psychosocial constructs such as perceived workplace support, which may be influenced by contextual factors and daily work experiences. The fair reliability observed may partly reflect the dynamic nature of IA, where fluctuations in symptoms such as pain or fatigue may influence individuals’ perceptions of support in the workplace. Although IA symptoms may fluctuate, workplace support from managers, coworkers and organizations is generally considered a relatively stable contextual factor over a short period. In addition, individuals on medically certified sick leave were excluded, reducing the likelihood that major health-related changes influenced responses between administrations.

The SEM and SDD values reported in the study (SDD ranging from 3.97 to 4.79) are within acceptable limits and consistent with other workplace support scales used in chronic disease populations [12, 14], indicating that the scale is sensitive enough to detect clinically meaningful changes. Minimal floor and ceiling effects across all subscales (ranging from 1% to 4.5%) demonstrate the scale’s ability to discriminate between various levels of perceived support without clustering at the extremes. This enhances the instrument’s responsiveness and its suitability for use in both clinical practice and research settings [21].

In addition to the main psychometric findings, descriptive data on participants’ confidence in working, mood, hand pain severity and overall health status offer valuable insight into their work-related experiences. The moderately high level of confidence in working reported by participants aligns with previous findings, suggesting that individuals with IA can maintain a positive perception of work ability when adequate support is present [12]. However, moderate levels of mood disturbance and hand pain severity observed in this sample suggest the presence of physical and emotional challenges that may impact job performance and long-term work sustainability [41]. Additionally, the general health perception being in the mid-range may reflect the fluctuating nature of inflammatory symptoms, which can influence both presenteeism and absenteeism [42, 43]. These contextual factors emphasize the importance of integrating psychosocial and ergonomic interventions into workplace support strategies for individuals with chronic musculoskeletal conditions.

This study has several limitations. First, although the sample was adequate for psychometric analysis, participants were recruited from a single university hospital, which may limit generalizability. Second, the test–retest interval was relatively short; future studies might consider assessing stability over longer periods and in response to workplace changes or interventions. The fair test–retest reliability suggests that the scale may be more suitable for research and group-level analyses rather than individual clinical monitoring. Third, detailed information about the cognitive demands of participants’ jobs, specific job titles or employment sectors was lacking. While physical demands were included because they are directly related to musculoskeletal symptoms in IA, cognitive load and work context may also influence perceptions of workplace support. Future studies should consider including a more comprehensive occupational profile that includes both physical and cognitive work demands and sectoral classifications to gain a more in-depth understanding of workplace factors affecting individuals with chronic conditions. Finally, the original developers of the instrument should review the veracity of the middle response option of ‘Do not agree or disagree’ and consider whether the option should be deleted from the presented choice of responses across all subscales.

## Conclusion

The Turkish version of the PWSS shows some supportive psychometric findings for assessing perceived workplace support among individuals with IA. It provides researchers and clinicians with a culturally appropriate tool to assess workplace dynamics that influence work engagement and support planning of people with chronic rheumatic conditions.

## Supplementary Material

rkag066_Supplementary_Data

## Data Availability

The data underlying this article will be shared on reasonable request to the corresponding author.
