# Supplementary material for: Turkish adaptation and psychometric testing of the Perceived Workplace Support Scale in people with inflammatory arthritis
Source: Rheumatol Adv Pract. 2026 Jun 9;10(3):rkag066. doi: 10.1093/rap/rkag066 (PMC13271243; doi:10.1093/rap/rkag066)
Supplement: rkag066_Supplementary_Data [file rkag066_supplementary_data.docx]

**Supplementary Data S1. Category Probability Curves of Item 1 “My manager does not understand…”**

**
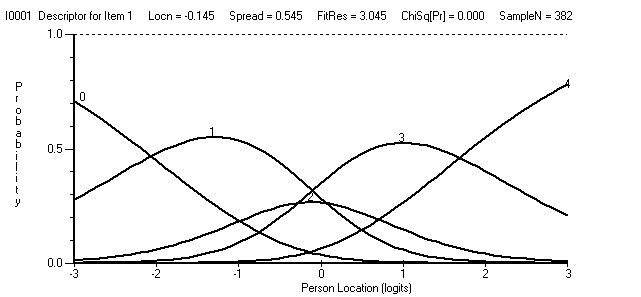
**

**ALT text:** The graph shows the probability of endorsing each of the five response categories (0–4) across the person ability continuum (logits). Each curve peaks where a response category is most likely to be selected.

**Supplementary Data S2. Category Probability Curves of Item 19 “The company/business organisation**

**tries to help employees make work arrangements that suit their personal needs.”**

**
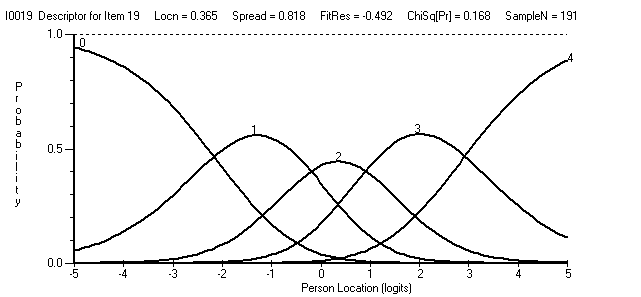
**

**ALT text:**

The graph shows the probability of endorsing each of the five response categories (0–4) across the person ability continuum (logits). Each curve peaks where a response category is most likely to be selected.

**Supplementary Data S3. Transformations**

**Manager**

| Raw Score | Manager |
| --- | --- |
| 0 | 0.0 |
| 1 | 2.5 |
| 2 | 4.2 |
| 3 | 5.3 |
| 4 | 6.0 |
| 5 | 6.5 |
| 6 | 6.9 |
| 7 | 7.3 |
| 8 | 7.6 |
| 9 | 8.0 |
| 10 | 8.4 |
| 11 | 8.9 |
| 12 | 9.5 |
| 13 | 10.4 |
| 14 | 11.7 |
| 15 | 13.5 |
| 16 | 16.0 |
|  |  |

**Co-Workers**

| Raw Score | Co-worker |
| --- | --- |
| 0 | 0.0 |
| 1 | 2.9 |
| 2 | 4.8 |
| 3 | 6.1 |
| 4 | 7.1 |
| 5 | 7.8 |
| 6 | 8.5 |
| 7 | 9.0 |
| 8 | 9.4 |
| 9 | 9.8 |
| 10 | 10.2 |
| 11 | 10.4 |
| 12 | 10.7 |
| 13 | 11.0 |
| 14 | 11.2 |
| 15 | 11.4 |
| 16 | 11.7 |
| 17 | 11.9 |
| 18 | 12.2 |
| 19 | 12.5 |
| 20 | 12.9 |
| 21 | 13.4 |
| 22 | 13.9 |
| 23 | 14.5 |
| 24 | 15.3 |
| 25 | 16.2 |
| 26 | 17.2 |
| 27 | 18.4 |
| 28 | 19.8 |
| 29 | 21.4 |
| 30 | 23.4 |
| 31 | 26.7 |
| 32 | 32.0 |

| **Company**  Raw Score | Company |
| --- | --- |
| 0 | 0.0 |
| 1 | 2.2 |
| 2 | 3.9 |
| 3 | 5.2 |
| 4 | 6.3 |
| 5 | 7.2 |
| 6 | 8.0 |
| 7 | 8.7 |
| 8 | 9.3 |
| 9 | 9.9 |
| 10 | 10.4 |
| 11 | 10.8 |
| 12 | 11.2 |
| 13 | 11.6 |
| 14 | 12.0 |
| 15 | 12.4 |
| 16 | 12.8 |
| 17 | 13.2 |
| 18 | 13.6 |
| 19 | 14.0 |
| 20 | 14.3 |
| 21 | 14.8 |
| 22 | 15.2 |
| 23 | 15.7 |
| 24 | 16.4 |
| 25 | 17.2 |
| 26 | 18.4 |
| 27 | 21.3 |
| 28 | 28.0 |

**Organisation**

| Raw Score | Organisation |
| --- | --- |
| 0 | 0.0 |
| 1 | 3.5 |
| 2 | 5.7 |
| 3 | 7.2 |
| 4 | 8.3 |
| 5 | 9.2 |
| 6 | 10.0 |
| 7 | 10.7 |
| 8 | 11.4 |
| 9 | 12.1 |
| 10 | 12.7 |
| 11 | 13.3 |
| 12 | 13.9 |
| 13 | 14.5 |
| 14 | 15.1 |
| 15 | 15.6 |
| 16 | 16.2 |
| 17 | 16.8 |
| 18 | 17.5 |
| 19 | 18.1 |
| 20 | 18.7 |
| 21 | 19.3 |
| 22 | 20.0 |
| 23 | 20.6 |
| 24 | 21.3 |
| 25 | 21.9 |
| 26 | 22.6 |
| 27 | 23.3 |
| 28 | 24.0 |
| 29 | 24.7 |
| 30 | 25.4 |
| 31 | 26.1 |
| 32 | 26.8 |
| 33 | 27.6 |
| 34 | 28.3 |
| 35 | 29.1 |
| 36 | 29.9 |
| 37 | 30.7 |
| 38 | 31.6 |
| 39 | 32.5 |
| 40 | 33.7 |
| 41 | 35.1 |
| 42 | 36.9 |
| 43 | 39.7 |
| 44 | 44.0 |

| \| **Total**  Raw Score \| Total \| \| --- \| --- \| \| 0 \| 0.0 \| \| 1 \| 5.1 \| \| 2 \| 8.3 \| \| 3 \| 10.4 \| \| 4 \| 11.9 \| \| 5 \| 13.1 \| \| 6 \| 14.1 \| \| 7 \| 15.1 \| \| 8 \| 15.9 \| \| 9 \| 16.7 \| \| 10 \| 17.5 \| \| 11 \| 18.2 \| \| 12 \| 18.9 \| \| 13 \| 19.6 \| \| 14 \| 20.2 \| \| 15 \| 20.9 \| \| 16 \| 21.5 \| \| 17 \| 22.1 \| \| 18 \| 22.7 \| \| 19 \| 23.3 \| \| 20 \| 23.9 \| \| 21 \| 24.4 \| \| 22 \| 25.0 \| \| 23 \| 25.5 \| \| 24 \| 26.1 \| \| 25 \| 26.6 \| \| 26 \| 27.1 \| \| 27 \| 27.6 \| \| 28 \| 28.2 \| \| 29 \| 28.7 \| \| 30 \| 29.2 \| \| 31 \| 29.7 \| \| 32 \| 30.2 \| \| 33 \| 30.7 \| \| 34 \| 31.2 \| \| 35 \| 31.7 \| \| 36 \| 32.2 \| \| 37 \| 32.7 \| \| 38 \| 33.2 \| \| 39 \| 33.8 \| \| 40 \| 34.3 \| \| 41 \| 34.8 \| \| 42 \| 35.4 \| \| 43 \| 35.9 \| \| 44 \| 36.5 \| \| 45 \| 37.1 \| \| 46 \| 37.7 \| \| 47 \| 38.3 \| \| 48 \| 38.9 \| \| 49 \| 39.5 \| \| 50 \| 40.2 \| \| 51 \| 40.8 \| \| 52 \| 41.5 \| \| 53 \| 42.2 \| \| 54 \| 42.9 \| \| 55 \| 43.6 \| \| 56 \| 44.4 \| \| 57 \| 45.1 \| \| 58 \| 45.9 \| \| 59 \| 46.7 \| \| 60 \| 47.5 \| \| 61 \| 48.4 \| \| 62 \| 49.2 \| \| 63 \| 50.1 \| \| 64 \| 51.0 \| \| 65 \| 52.0 \| \| 66 \| 53.0 \| \| 67 \| 54.0 \| \| 68 \| 55.1 \| \| 69 \| 56.3 \| \| 70 \| 57.6 \| \| 71 \| 59.2 \| \| 72 \| 60.9 \| \| 73 \| 63.1 \| \| 74 \| 65.9 \| \| 75 \| 70.0 \| \| 76 \| 76.0 \| |  |
| --- | --- | --- | --- | --- | --- | --- | --- | --- | --- | --- | --- | --- | --- | --- | --- | --- | --- | --- | --- | --- | --- | --- | --- | --- | --- | --- | --- | --- | --- | --- | --- | --- | --- | --- | --- | --- | --- | --- | --- | --- | --- | --- | --- | --- | --- | --- | --- | --- | --- | --- | --- | --- | --- | --- | --- | --- | --- | --- | --- | --- | --- | --- | --- | --- | --- | --- | --- | --- | --- | --- | --- | --- | --- | --- | --- | --- | --- | --- | --- | --- | --- | --- | --- | --- | --- | --- | --- | --- | --- | --- | --- | --- | --- | --- | --- | --- | --- | --- | --- | --- | --- | --- | --- | --- | --- | --- | --- | --- | --- | --- | --- | --- | --- | --- | --- | --- | --- | --- | --- | --- | --- | --- | --- | --- | --- | --- | --- | --- | --- | --- | --- | --- | --- | --- | --- | --- | --- | --- | --- | --- | --- | --- | --- | --- | --- | --- | --- | --- | --- | --- | --- | --- | --- | --- | --- | --- | --- |
